# Neural Correlates of Age-Related Changes in Precise Grip Force Regulation: A Combined EEG-fNIRS Study

**DOI:** 10.3389/fnagi.2020.594810

**Published:** 2020-12-10

**Authors:** Alisa Berger, Fabian Steinberg, Fabian Thomas, Michael Doppelmayr

**Affiliations:** ^1^Department of Sport Psychology, Institute of Sport Science, Johannes Gutenberg-University Mainz, Mainz, Germany; ^2^School of Kinesiology, Louisiana State University, Baton Rouge, LA, United States; ^3^Centre for Cognitive Neuroscience, Paris Lodron University of Salzburg, Salzburg, Austria

**Keywords:** electroencephalography, functional near-infrared spectroscopy, aging, motor control, motor recovery, neuroplasticity

## Abstract

Motor control is associated with suppression of oscillatory activity in alpha (8–12 Hz) and beta (12–30 Hz) ranges and elevation of oxygenated hemoglobin levels in motor-cortical areas. Aging leads to changes in oscillatory and hemodynamic brain activity and impairments in motor control. However, the relationship between age-related changes in motor control and brain activity is not yet fully understood. Therefore, this study aimed to investigate age-related and task-complexity-related changes in grip force control and the underlying oscillatory and hemodynamic activity. Sixteen younger [age (mean ± SD) = 25.4 ± 1.9, 20–30 years] and 16 older (age = 56.7 ± 4.7, 50–70 years) healthy men were asked to use a power grip to perform six trials each of easy and complex force tracking tasks (FTTs) with their right dominant hand in a randomized within-subject design. Grip force control was assessed using a sensor-based device. Brain activity in premotor and primary motor areas of both hemispheres was assessed by electroencephalography (EEG) and functional near-infrared spectroscopy (fNIRS). Older adults showed significantly higher inaccuracies and higher hemodynamic activity in both FTTs than did young adults. Correlations between grip force control owing to task complexity and beta activity were different in the contralateral premotor cortex (PMC) between younger and older adults. Collectively, these findings suggest that aging leads to impairment of grip force control and an increase in hemodynamic activity independent of task complexity. EEG beta oscillations may represent a task-specific neurophysiological marker for age-related decline in complex grip force control and its underlying compensation strategies. Further EEG-fNIRS studies are necessary to determine neurophysiological markers of dysfunctions underlying age-related motor disabilities for the improvement of individual diagnosis and therapeutic approaches.

## Introduction

The ability to adapt our actions quickly to changes in the environment is a key function of human motor skills (Hermsdörfer et al., 2003). One of the most sophisticated characteristics of fine motor skills is the precise grip force regulation according to the physical requirements of a manipulated object, which is necessary for the successful performance of various everyday activities (Hermsdörfer et al., 2003; Voelcker-Rehage and Alberts, 2005; Parry et al., 2019). When gripping an object, radial forces are exerted that are large enough to prevent the object from slipping while ensuring that it does not break. Tangential forces are applied to move the object along the desired path (Flanagan and Wing, 1995; Haggard and Wing, 1995). Thus, regulation of precise grip force requires the coupling of radial and tangential grip forces (Hermsdörfer et al., 2000; Nowak et al., 2001; Rand et al., 2004), which is based on visuomotor transformation processes and the intact integration of sensory feedback within the motor-cortical network (Hermsdörfer et al., 2003; Prodoehl et al., 2009). Functional activity in motor-cortical areas can be quantified as cerebral hemodynamics using brain imaging techniques such as functional magnetic resonance imaging (fMRI) and functional near-infrared spectroscopy (fNIRS; Agbangla et al., 2017) or as electrical signals using the electroencephalography (EEG; Hamacher et al., 2015). It is suggested that synchronous oscillatory activity in the alpha (8–12 Hz) and beta (12–30 Hz) ranges within the motor-cortical network represents the basic mechanism for functional communication underlying the motor control process (Davis et al., 2012; Wach et al., 2013; Pollok et al., 2015). The alpha rhythm reflects cortical processes during the awake and attentive-resting states, and the amplitude of the signal is suppressed by sensory stimulation or during the motor control process (Engel and Fries, 2010). Beta oscillations are attenuated during voluntary movements, which is known as movement-related beta desynchronization (MRBD; Zaepffel et al., 2013). After the completion of a movement, beta oscillations are more pronounced, which has been interpreted as increased inhibition to maintain the status quo of the motor system (Pfurtscheller et al., 2006; Koelewijn et al., 2008; Engel and Fries, 2010). Previous studies have shown that oscillatory activity and cerebral hemodynamics are inversely related during the motor control process, that is, hemodynamic activity increases with a decrease in alpha and beta oscillations (Lachert et al., 2017). Changes in brain activity during the motor control process have been observed in various cerebral structures (Zaepffel et al., 2013), mainly in sensorimotor areas with a contralateral predominance (Taniguchi et al., 2000). Concerning grip force tasks, as an example, young healthy people exhibited increased hemodynamic activity in the sensorimotor cortex (SMC) and in the premotor cortex (PMC; Ehrsson et al., 2000; Cramer et al., 2002; Wriessnegger et al., 2017). The SMC contralateral to the active hand showed the highest hemodynamic activity (Wriessnegger et al., 2017) confirming the involvement of the SMC during movement execution (Leff et al., 2011). Furthermore, the prefrontal cortex (PFC), supplementary motor area (SMA), and PMC bilaterally as well as the cerebellum were found to be involved in grip force execution (Dai et al., 2001). Thereby, the SMA represents a key structure controlling the motor-cortical network while driving the regulation of grip forces by promoting and suppressing its activity (Grefkes et al., 2008a). Alterations in brain functions within the motor-cortical network can be caused by neurological diseases (Schnitzler and Gross, 2005; Grefkes et al., 2008b; Engel and Fries, 2010; Kiyama et al., 2014). According to previous studies, reduced inter-and intra-hemispheric connectivity between the SMA and the primary motor cortex (Grefkes et al., 2008b), upregulation of the parieto-frontal network activity in the stroke-lesioned hemisphere (Bönstrup et al., 2018), and pathological increases in beta oscillations (Rossiter et al., 2014a) were correlated significantly with motor deficits (Grefkes et al., 2008b; Rossiter et al., 2014a; Bönstrup et al., 2018).

More than one billion people worldwide suffer from neurological diseases. The number will increase in the coming years because of the aging population (Semprini et al., 2018). Early signs of age-related diseases are altered brain structures and synaptic functions ranging from pathological oscillations and altered functional connectivity to reduced gray matter volume (Minati et al., 2007; Ishii et al., 2017; Xifra-Porxas et al., 2019). During grip force control, older adults exhibited higher beta power, greater (more negative) MRBD (Xifra-Porxas et al., 2019), and significantly higher hemodynamic activity in subcortical areas and PMC than did young healthy adults (Noble et al., 2011). Deficits in fine motor skills and behavioral declines are most often the consequences of these changes (Voelcker-Rehage and Alberts, 2005; Grefkes et al., 2008b; Engel and Fries, 2010; Kiyama et al., 2014). However, the nature of the relationship between age-related changes in brain activity patterns and declines in grip force regulation has not been fully elucidated. Previous studies concerning age-related deteriorations in motor functions used tasks such as finger tapping (Naccarato et al., 2006), key pressing (Mattay et al., 2002), index finger abductions/adductions, or wrist extensions/flexions (Hutchinson et al., 2002), thus focusing on repetitive movements that are limited in elderly individuals, when activities of everyday lives that require the regulation of grip forces must be performed (Diermayr et al., 2011; Bock and Steinberg, 2012). It is, therefore, important to investigate how the precise regulation of grip forces is related to brain activity and is influenced by the aging process.

In this study, EEG and fNIRS, which are well-established, promising electro/neurophysiological techniques that are affordable, easy to implement, and easy to integrate into the diagnosis of neurological conditions in patients of all age groups, were used (Makeig et al., 2009; Herold et al., 2017). Knowledge of how grip force regulation and hemodynamic/oscillatory brain activity are related and altered according to age provides crucial information for individualization of therapy protocols or advancement of interventions such as neurofeedback, brain-machine interfaces, or noninvasive brain stimulation (NIBS; Gassert and Dietz, 2018; Semprini et al., 2018; Berger et al., 2019a). More specifically, for example, identifying pathological hypo-/hyperactivity or changes in oscillatory activity while applying fine motor skills could guide future NIBS or training protocols to enable the administration of target-oriented and individualized interventions (Teo et al., 2016; Berger et al., 2018; Gassert and Dietz, 2018).

Therefore, the central purpose of this study was to investigate age- and task-related changes in hemodynamic and oscillatory activity during two visually guided force tracking tasks (FTTs) of different complexities. The easy FTT requires static grip force, whereas the complex FTT requires grip force-related characteristics necessary in everyday life settings (e.g., fine-tuning and quick adjustments of grip forces according to changing demands, such as when grasping objects to prevent slipping). As discussed above and based on the findings of previous studies that focused on task complexity (Holper et al., 2009; Wriessnegger et al., 2017) or age-related changes (Voelcker-Rehage and Alberts, 2005; Noble et al., 2011; Rossiter et al., 2014a,b; Xifra-Porxas et al., 2019), we hypothesized differences between the behavioral and neuronal factors associated with the two FTTs and between the younger and older adults (Noble et al., 2011) as follows: we expected: (1) that the complex FTT, which requires the regulation of grip force, results in higher inaccuracies in grip force control and greater brain activity in motor-cortical areas than does the easy FTT; and (2) that the older adults show greater deficits in precise grip force control and changes in sensorimotor activity during complex FTT than did younger adults.

## Materials and Methods

This study was performed following the ethical standards laid down in the Declaration of Helsinki. Experimental procedures were performed according to the recommendations of the Deutsche Gesellschaft für Psychologie (DGP) and approved by the local ethics committee of the Johannes Gutenberg-Universität Mainz. All participants were informed about the study-related contents before obtaining written informed consent before the initiation of the experiment.

### Participants

Sixteen younger male participants aged 20–30 years (mean age: 25.44 ± 1.93 years) and sixteen older male participants aged 50–70 years (mean age: 56.75 ± 4.71 years) without any hand pathologies and extremity injuries were recruited to participate in this study. All participants were right-handed according to the Edinburg Handedness-scale (Oldfield, 1971), had no neurological or psychological disorders, and had a normal or corrected-to-normal vision. They were asked to disclose information on preexisting neurological, psychological, and medical conditions and on drug and alcohol/caffeine intake during the previous week.

### Experimental Equipment and Data Acquisition

#### Grip Force Measures

**Figure 1:**
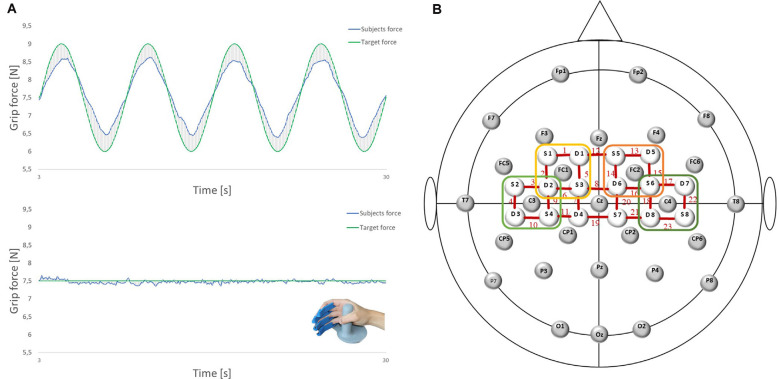
**(A)** Force tracking tasks (FTTs) and the force transducer for the right dominant hand (LIME medical GmbH, Germany): complex FTT (top); easy FTT (bottom); green: target force; blue: subjects force.** (B)** Electroencephalography (EEG)/functional near-infrared spectroscopy (fNIRS) Montage: 28 EEG electrodes and 16 fNIRS optodes placed according to the 5:10 EEG system. Regions of interest are marked. Left premotor cortex (PMC): FC1 and Channels 1, 2, 5, 6 (yellow); right PMC: FC2 and Channels 13, 14, 15, 16 (orange); left sensorimotor cortex (SMC): C3 and Channels 3, 4, 9, 10 (light green); right SMC: C4 and Channels 17, 18, 2, 23 (dark green).

The ability of precise grip force control was measured using the FTT, which is a well-known diagnostic tool used to investigate specific aspects of force control, that is, force generation and force release (Voelcker-Rehage and Alberts, 2005). It consists of a monitor and a force transducer for the dominant right hand (LIME medical GmbH, Germany). Four rails for the index, middle, ring, and little fingers guarantee a standardized position of the hand (see Figure 1A). The participants were instructed to use the power grip to match a curve representing the applied force of their dominant (right) hand to a predefined target route as accurately as possible. The participants and the target route were displayed on a screen at approximately 80 cm. The participants aimed to make as few deviations as possible. A deviation is defined as the absolute difference between the applied force and the optimal target force. All forces (the participant’s applied grip force; predefined, optimal target force; and the resulting deviation) were measured in Newton (N) and calculated with a sampling rate of 10 Hz.

#### EEG Measures

EEG was recorded using Brain Vision Recorder 1.2 Brain Products, Germany equipped with 32 active Ag/AgCl electrodes, at a sampling rate of 1,000 Hz (notch filter at 50 Hz). Head dimensions were measured individually, and caps were adapted to the corresponding head sizes. Data were recorded using 28 electrodes placed on the scalp at Fp1, Fp2, F7, F3, Fz, F4, F8, FC5, FC1, FC2, FC6, T7, C3, Cz, C4, T8, CP5, CP1, CP2, CP6, P7, P3, Pz, P4, P8, O1, Oz, and O2 according to the international 5:10 system (Jurcak et al., 2007; see Figure 1B). Vertical and horizontal electrooculograms were recorded with four additional electrodes placed next to the left and right eye and above/below the right eye to detect eye movements and eyelid artifacts. The reference and ground electrodes were placed at the nose tip and AFz location, respectively. Impedance was maintained below 5 kΩ by using a SuperViscTM electrode filled with gel (EASYCAP GmbH, Germany) for conductivity.

#### fNIRS Measures

For fNIRS measurement, cerebral oxygenation changes were recorded using a near-infrared optical tomographic imaging device (NIRSport, NIRx, Germany, Wavelengths: 760 nm, 850 nm, sampling rate: 7.81 Hz). The methodology and underlying physiology are explained in detail elsewhere (Obrig and Villringer, 2003). A total of 16 optodes (eight emitters, eight detectors) were placed at 3 cm intervals above the motor cortex according to the international 5:10 system (Jurcak et al., 2007), resulting in 23 channels (source-detector pairs; see Figure 1B). Channel positions covered identical regions of both hemispheres, including the SMC [Brodmann Area (BA) 1–4] and SMA/PMC (BA6).

#### Experimental Design and Procedure

To control the maximum voluntary contraction (MVC) of the participants, a standardized Grip-Strength-Test was performed before the experiment. Participants sat upright in a standardized position on a height-adjustable chair (Massy-Westropp et al., 2011). The right upper arm was abducted, the elbow joint was flexed at 90°, the wrist was in a neutral zero position and the arm was held close to the body but not resting on the armrest of the chair. Using an electronic hand dynamometer (TL-LSC100, Trailite), three values of MVC were measured with the dominant right hand. The rest period in between was 1 min.

**Figure 2:**
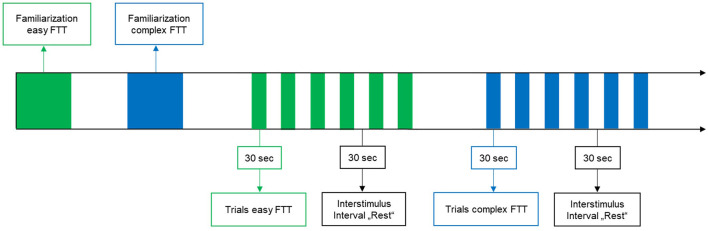
Study design.

Participants performed two visually-guided grip-FTTs in a randomized order: in one task (easy FTT), participants had to follow a target force depicted as a straight line at 7.5 N by maintaining a constant grip force with the right dominant hand. In the other task (complex FTT), the target force changed over time in the form of a sinusoidal curve averaged to 7.5 N (6–9 N). Participants had to regulate and adapt their grip forces (N) continuously to the target value (see Figure 1A). Each FTT consisted of six task trials, with each trial lasting 30 s and alternating with six trials of rest (30 s; see Figure 2). During the trials of rest, participants were instructed to watch a fixation cross on the screen. Before and after each FTT, brain activity was recorded at rest for 30 s each with eyes closed and open. During the measurements, participants were instructed to avoid head movements and talking to reduce motion and physiological artifacts (Vitorio et al., 2017). For familiarization, two trials of the easy FTT and two trials of the complex FTT were performed before the experiment.

### Data Processing

#### Grip Force Measures

For the MVC, the mean of the three values was collected from each participant. Then, the arithmetic mean was calculated for the younger and older adults. Regarding the FTT, the participant’s applied grip force, the predefined, optimal target force, and their absolute deviations were measured for each FTT trial. Data from the first 3 s of each trial were excluded from further analysis considering the initial force adaptation at the onset. Then, the arithmetic mean of the absolute deviation values was computed, which represents the inaccuracy in grip force control (Gölz et al., 2018). Normal distribution was examined using the Shapiro–Wilk test (*p* ≥ 0.05). A two-way repeated-measures analysis of variance (rmANOVA) using the factors TASK (easy FTT, complex FTT) and GROUP (younger adults, older adults) was conducted to evaluate changes in grip force control owing to task complexity and aging. Bonferroni-adjusted *post-hoc* analyses were used to identify significant differences.

#### EEG Measures

Electrophysiological data were preprocessed using the Brain Vision Analyzer 2.0 (Brain Products, Gilching, Germany) by applying a 0.5 Hz high-pass filter and a 50 Hz low-pass filter. Horizontal and vertical eye movements were corrected using the Gratton and Coles methods (Gratton et al., 1983). Any remaining artifacts were removed using a semiautomatic inspection tool by setting the maximum allowed gradient voltage step at 50 mV/ms and the maximally allowed amplitude at 100 mV. Further artifacts were visually identified and manually rejected so that only segments containing no artifacts were included for further processing. For the evaluation of changes in oscillatory activity over time, the two task conditions (easy FTT and complex FTT) and the rest condition were subdivided into six trials of 30 s. Each condition was segmented into 1 s epochs, a fast Fourier transformation [Maximum Resolution (0.977 Hz); Power (μV^2^); No window] was computed, and frequency spectra of the artifact-free segments were averaged for each condition. Then, oscillatory mean activities (mV) were calculated for the alpha (8–12 Hz) and beta (13–30 Hz) ranges. Since suppressed beta activity relative to rest is associated with movement execution (Xifra-Porxas et al., 2019), the MRBD was calculated in addition to the absolute power. Therefore, beta activity at rest as well as the difference of beta activity between each FTT and rest was calculated and statistically analyzed. Following the fNIRS channels and the corresponding regions of interest (ROI), the following EEG electrodes were used for further statistical analysis: left SMA/PMC, FC1; right SMA/PMC, FC2; left SMC, C3; right SMC: C4 (see Figure 1B).

#### fNIRS Measures

Raw brain oxygenation data were preprocessed and analyzed using the time series analysis routine within the MATLAB-based NIRSlab analysis package (v2017.05, Nirx Medical Technologies, Glen Head, NY, USA; Xu et al., 2014) according to the current recommendations (Herold et al., 2017; Vitorio et al., 2017). Concentration changes of oxygenated hemoglobin (Hboxy) and deoxygenated hemoglobin (Hbdeoxy) were visually inspected concerning transient spikes and abrupt discontinuities representing the two most common forms of movement artifacts in fNIRS data. To achieve smoothening, epochs that contain discontinuities (or “jumps”) or long-term drifts were corrected, and spikes were replaced by the nearest signal (Xu et al., 2014). Then, a band-pass filter (0.01–0.2 Hz) was applied to remove slow drifts and high frequencies related to breathing, respiratory or cardiac rhythm, vasomotion, or other movement artifacts (Koenraadt et al., 2014). After preprocessing, parameters such as wavelengths (WL1 = 760 nm; WL2 = 850 nm), differential pathlength factors (7.25 for WL1; 6.38 for WL2), interoptode distances (3 cm), and background tissue values (totHb: 75 μM; MVO2Sat: 70%) were specified, and the time series of Hboxy/Hbdeoxy concentration changes (ΔHboxy/ΔHboxy) were computed using the modified Beer-Lambert law (Cope et al., 1988; Sassaroli and Fantini, 2004). Afterward, ΔHboxy data of the easy and complex FTT trials (30 s) were exported to analyze their hemodynamic responses. Averaged baseline concentration values of 15 s rest before each trial were subtracted from the task-evoked concentration measurements to account for time-dependent changes in cerebral oxygenation (Vitorio et al., 2017). ΔHboxy was calculated for ROI (left SMA/PMC: Channels 1, 2, 5, 6; right SMA/PMC: Channels 13, 14, 15, and 16; left SMC: Channels 3, 4, 9, and 10; right SMC: Channels: 17, 18, 22, 23) during the FTTs and was used as a marker for regional cortical activation because it is more sensitive to motor-related activities than is Hbdeoxy (Suzuki et al., 2004) and because it is an accurate indicator of hemodynamic activity (Strangman et al., 2002).

#### Statistical Analysis

The statistical analysis of data corresponding to oscillatory and hemodynamic activity was conducted using SPSS 23 (IBM, Armonk, NY, USA). The normality of distribution was examined using the Shapiro–Wilk test (*p* ≥ 0.05). Oscillatory mean activities and averaged ΔHboxy values corresponding to both the FTTs were computed for each subject and ROI (Herold et al., 2017; Vitorio et al., 2017). A three-way rmANOVA with the factors TASK (easy FTT, complex FTT), ROI [ROI1: left SMA/PMC (BA6); ROI2: right SMA/PMC (BA6); ROI3: left SMC (BA1–4); ROI4: right SMC (BA1–3)], and GROUP (younger adults, older adults) was conducted to evaluate changes in oscillatory and hemodynamic activity owing to task complexity, brain regions, and between the groups. In case of significant effects, Bonferroni-adjusted *post-hoc* analyses were used to identify significant differences between the tasks, ROI, and groups.

The overall level of significance was set to *p* ≤ 0.05. Significant tendencies were defined as *p* ≤ 0.10. Mauchly’s test was used to check for any violations of sphericity. If a violation of sphericity was detected (*p* ≤ 0.05) and a Greenhouse–Geisser ε > 0.75 existed, Huynh-Feldt corrected *p*-values were reported. Otherwise (ε ≤ 0.75), a Greenhouse-Geisser correction was applied. Effect sizes were reported in the partial eta-square ($\eta_{\text{p}}^{2}$) for the significant main effects and interactions. According to Cohen (1988), we classified the effect sizes as follows: >0.01 for small, >0.06 for medium, and >0.14 for large effect sizes (Cohen, 1988).

To calculate the relationship between precise grip force control, oscillatory and hemodynamic activity, correlation analysis was performed for each task, region, and group. First, correlations were calculated for each task separately (e.g., correlation of inaccuracy and beta activity during the easy FTT). Second, differences between the easy FTT and the complex FTT were calculated. Then, difference values (Δ = complex FTT − easy FTT) were correlated (e.g., correlation of Δinaccuracy and Δβ activity) to analyze task complexity-related effects. Degrees of correlation between grip force control, oscillatory and hemodynamic activity were explored using the Pearson correlation coefficient: low, *r* > 0.1–0.3; medium, *r* > 0.3–0.5; strong, *r* > 0.5–0.7; very strong, *r* > 0.7–0.9; or perfect, *r* > 0.9. Furthermore, the correlations of younger and older adults were statistically compared (Eid et al., 2015).

## Results

### Grip Force Measures

**Figure 3:**
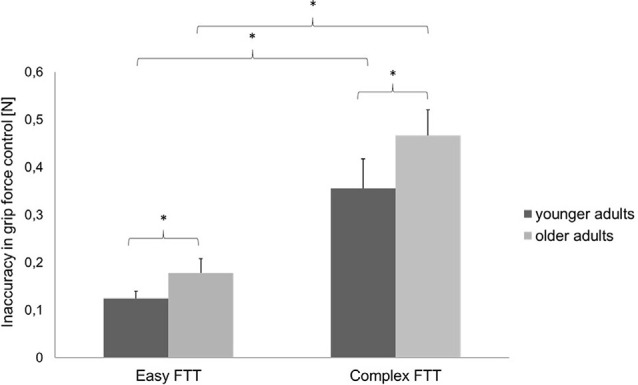
Precision in grip force control (mean ± SD) of the younger and older adults during the easy and complex FTTs; *p < 0.05.

Regarding the MVC, no differences between the two groups were presented (younger adults: 45.45 ± 4.43, younger adults: 45.59 ± 7.67, *p* = 0.951). For the FTT, the two-way repeated-measures ANOVA (rmANOVA) using the factors TASK (easy FTT, complex FTT) and GROUP (younger adults, older adults) showed a statistically significant main effect of TASK (*F*_(1,30)_ = 184.10, *p* < 0.001, $\eta_{\text{p}}^{2}$ = 0.86) that indicates the incidence of a significantly higher inaccuracy during the complex FTT than during the easy FTT (*p* < 0.001). Furthermore, a significant main effect of GROUP (*F*_(1,30)_ = 6.51, *p* = 0.02, $\eta_{\text{p}}^{2}$ = 0.18) emerged that presented higher inaccuracies in grip force regulation in older adults than in young adults (*p* = 0.02). No significant interaction of TASK × GROUP was observed (*F*_(1,30)_ = 2.24, *p* = 0.15, $\eta_{\text{p}}^{2}$ = 0.07; see Figure 3).

### fNIRS Measures

**Figure 4:**
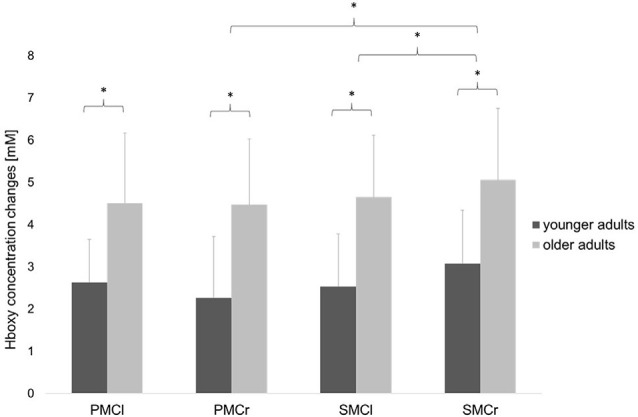
Relative concentration changes of Hboxy (mean ± SD) in the four regions of interest averaged over the easy FTT and the complex FTT for the younger and older adults. *p < 0.05.

Statistical analysis of data on relative Hboxy concentration changes revealed a significant main effect of GROUP (*F*_(1,30)_ = 4.36, *p* = 0.04, $\eta_{\text{p}}^{2}$ = 0.13), indicating a significantly higher hemodynamic activity in the older adults than in the young adults. The significant main effect of ROI (*F*_(3,90)_ = 3.56, *p* = 0.02, $\eta_{\text{p}}^{2}$ = 0.11) showed significant differences between the cortical areas. Bonferroni-corrected *post-hoc* tests revealed significantly higher hemodynamic activity in the ipsilateral SMC than in the ipsilateral PMC (*p* = 0.04) and contralateral SMC (*p* = 0.01; see Figure 4). Otherwise, no further main effects or interactions were observed (all *p* > 0.13).

### EEG Measures

**Figure 5:**
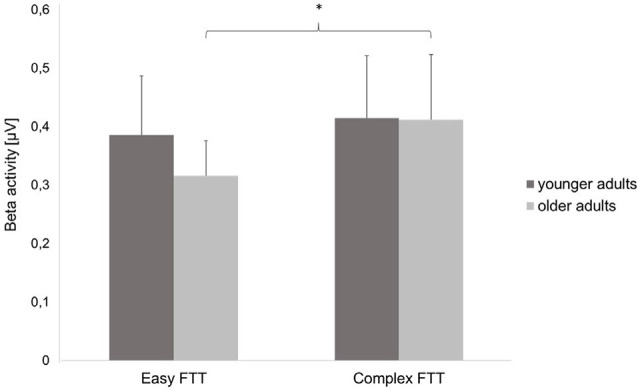
Beta activity (mean ± SD) of the younger and older adults during the easy and complex FTT, *p < 0.05.

**Figure 6:**
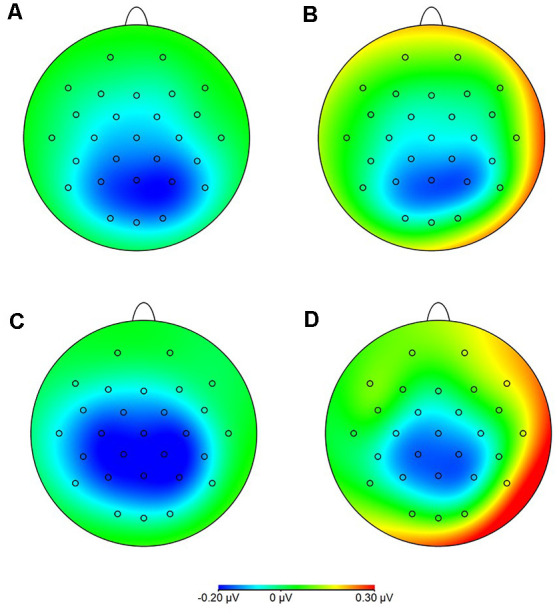
Movement-related beta desynchronization (MRBD) **(A)** younger adults, easy FTT **(B)** younger adults, complex FTT **(C)** older adults, easy FTT **(D)** older adults, complex FTT.

Statistical analysis of data on oscillatory alpha activity (8–12 Hz) using a three-way rmANOVA with the factors TASK, ROI, and GROUP revealed neither main effects nor interaction effects (all *p* > 0.10). Concerning beta activity (13–30 Hz), the three-way rmANOVA showed a significant main effect of TASK (*F*_(1,30)_ = 11.23, *p* < 0.001, $\eta_{\text{p}}^{2}$ = 0.27), indicating a significantly higher beta activity during the complex FTT than during the easy FTT. The significant main effect ROI (*F*_(3,90)_ = 5.65, *p* = 0.02, $\eta_{\text{p}}^{2}$ = 0.16) revealed significantly higher beta activity in the left SMC than in the PMC and bilateral SMC. Furthermore, a statistically significant trend for the interaction of TASK × GROUP (*F*_(1,30)_ = 3.10, *p* = 0.08, $\eta_{\text{p}}^{2}$ = 0.09) emerged. Bonferroni-corrected *post-hoc* tests indicated significantly higher beta activity during the complex FTT than during the easy FTT in the group of older adults (*p* = 0.001; see Figure 5). Furthermore, analysis of data on beta activity during rest, the two-way rmANOVA with the factors ROI, and GROUP revealed neither main effects nor interaction effects (all *p* > 0.10). Regarding the MRBD, the three-way rmANOVA showed a significant main effect of TASK (*F*_(1,30)_ = 11.04, *p* < 0.002, $\eta_{\text{p}}^{2}$ = 0.27), indicating a significantly greater (more negative) MRBD during the easy FTT than during the complex FTT. The significant main effect ROI (*F*_(3,90)_ = 5.41, *p* = 0.01, $\eta_{\text{p}}^{2}$ = 0.15) revealed significantly higher beta suppression in the left SMC than in the left PMC. Furthermore, a statistically significant trend for the interaction of TASK × GROUP (*F*_(1,30)_ = 3.11, *p* = 0.08, $\eta_{\text{p}}^{2}$ = 0.09) and TASK × ROI × GROUP (*F*_(3,90)_ = 2.78, *p* = 0.08, $\eta_{\text{p}}^{2}$ = 0.09) emerged. Bonferroni-corrected *post-hoc* tests indicated that older adults exhibited significantly greater MRBD on the SMC bilaterally during the easy FTT compared to the younger adults (left SMC *p* = 0.04, right SMC *p* = 0.03; see Figure 6).

### Associations Between Precise Grip Force Control, Oscillatory and Hemodynamic Activity

**Figure 7:**
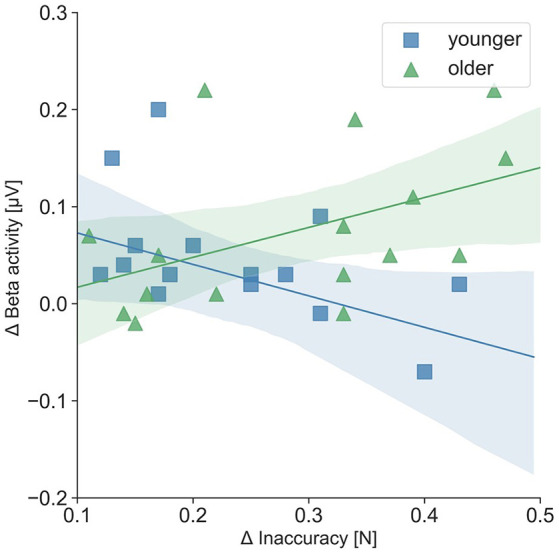
Correlation of Δ inaccuracy in precise grip force regulation and Δ left PMC beta activity (μV); Δ = complex FTT − easy FTT; younger adults: r = − 0.32; older adults: *r* = 0.46. The correlations differed significantly between younger and older adults (*p* = 0.03).

For the separate analysis of the tasks, correlations between precise grip force control and oscillatory activity as well as precise grip force control and hemodynamic activity were statistically not significant. Regarding the relation of oscillatory beta activity and hemodynamic activity, three significant tendencies (*p* < 0.08) and one significant correlation of beta activity in the right SMC and hemodynamic activity in the left SMC emerged during the easy FTT for the younger adults (*r* = 0.55, *p* = 0.03). For the older adults, correlation analysis showed nine significant tendencies (*p* < 0.08) and one significant correlation of beta activity in the right PMC and hemodynamic activity in the right SMC during the easy FTT (*r* = 0.50, *p* = 0.05; see Table 1). During the complex FTT, no significant tendencies or correlations were presented. Concerning complexity-based differences (Δ = complex FTT − easy FTT), older adults showed a positive association with a trend between inaccuracy in precise grip force regulation and oscillatory beta activity in the left PMC (*r* = 0.46, *p* = 0.07), whereas young adults showed a non-significant negative association (*r* = − 0.32, *p* = 0.22; see Figure 7). However, these correlations differed significantly between younger and older adults (*p* = 0.03). Thus, complexity-related deteriorations are accompanied by different motor cognitive processes in young and older adults.

**Table 1:** Correlations between relative oxygenated hemoglobin (Hboxy) and oscillatory beta activity.

ROI	EEG left PMC	EEG right PMC	EEG left SMC	EEG right SMC
Easy FTT—younger adults
fNIRS left PMC	*r* = 0.16 (*p* = 0.552)	*r* = 0.30 (*p* = 0.635)	*r* = 0.16 (*p* = 0.543)	*r* = 0.28 (*p* = 0.297)
fNIRS right PMC	*r* = 0.30 (*p* = 0.250)	*r* = 0.28 (*p* = 0.283)	*r* = 0.33 (*p* = 0.206)	*r* = 0.43 (*p* = 0.099)
fNIRS left SMC	*r* = 0.45 (*p* = 0.080)	*r* = 0.43 (*p* = 0.096)	*r* = 0.46 (*p* = 0.071)	*r* = 0.55 (*p* = 0.027)*
fNIRS right SMC	*r* = 0.37 (*p* = 0.160)	*r* = 0.34 (*p* = 0.197)	*r* = 0.37 (*p* = 0.151)	*r* = 0.37 (*p* = 0.062)
Easy FTT—older adults
fNIRS left PMC	*r* = 0.41 (*p* = 0.110)	*r* = 0.46 (*p* = 0.076)	*r* = 0.43 (*p* = 0.098)	*r* = 0.26 (*p* = 0.335)
fNIRS right PMC	*r* = 0.44 (*p* = 0.087)	*r* = 0.47 (*p* = 0.065)	*r* = 0.48 (*p* = 0.083)	*r* = 0.26 (*p* = 0.325)
fNIRS left SMC	*r* = 0.47 (*p* = 0.069)	*r* = 0.49 (*p* = 0.055)	*r* = 0.47 (*p* = 0.066)	*r* = 0.25 (*p* = 0.351)
fNIRS right SMC	*r* = 0.48 (*p* = 0.062)	*r* = 0.50 (*p* = 0.048)*	*r* = 0.47 (*p* = 0.064)	*r* = 0.24 (*p* = 0.368)
Complex FTT—younger adults
fNIRS left PMC	*r* = 0.12 (*p* = 0.661)	*r* = −0.14 (*p* = 0.615)	*r* = −0.20 (*p* = 0.662)	*r* = 0.12 (*p* = 0.964)
fNIRS right PMC	*r* = −0.07 (*p* = 0.791)	*r* = −0.09 (*p* = 0.730)	*r* = −0.34 (*p* = 0.888)	*r* = 0.07 (*p* = 0.800)
fNIRS left SMC	*r* = 0.12 (*p* = 0.654)	*r* = 0.09 (*p* = 0.730)	*r* = 0.15 (*p* = 0.590)	*r* = 0.25 (*p* = 0.348)
fNIRS right SMC	*r* = 0.01 (*p* = 0.963)	*r* = −0.18 (*p* = 0.948)	*r* = 0.03 (*p* = 0.914)	*r* = 0.16 (*p* = 0.554)
Complex FTT—older adults
fNIRS left PMC	*r* = 0.33 (*p* = 0.207)	*r* = 0.36 (*p* = 0.166)	*r* = 0.36 (*p* = 0.176)	*r* = 0.27 (*p* = 0.310)
fNIRS right PMC	*r* = 0.36 (*p* = 0.172)	*r* = 0.40 (*p* = 0.122)	*r* = 0.39 (*p* = 0.141)	*r* = 0.30 (*p* = 0.265)
fNIRS left SMC	*r* = 0.37 (*p* = 0.157)	*r* = 0.36 (*p* = 0.169)	*r* = 0.38 (*p* = 0.148)	*r* = 0.25 (*p* = 0.358)
fNIRS right SMC	*r* = 0.40 (*p* = 0.126)	*r* = 0.38 (*p* = 0.138)	*r* = 0.40 (*p* = 0.134)	*r* = 0.25 (*p* = 0.361)

Abbreviations: PMC, premotor cortex; SMC, sensorimotor cortex; *r* = Pearson correlation coefficient; Significance level *p* < 0.05*.

## Discussion

This study aimed to investigate age-related changes in grip force regulation and associated changes in oscillatory and hemodynamic brain activity. The following is the summary of our findings: (1) age-related deteriorations in grip force control and increases in hemodynamic activity in motor-cortical areas occurred independently of task complexity and (2) deteriorations in grip force control owing to the task complexity was positively associated with left PMC beta activity in older adults and negatively associated in younger adults.

First, the older adults showed significantly greater inaccuracies in grip force control and significantly higher hemodynamic brain activity in all motor-cortical areas during the easy and complex FTTs than did the younger adults. These results confirm the existence of expected age-related deteriorations in grip force control (Voelcker-Rehage and Alberts, 2005) and increases in hemodynamic activity (Mattay et al., 2002; Naccarato et al., 2006; Kim et al., 2010; Noble et al., 2011). On one hand, aging is associated with diminished tactile sensations that reduce hand sensibilities, which lead to declines in fine motor skills (Cole, 1991; Bennett and Castiello, 1994; Voelcker-Rehage and Alberts, 2005). On the other hand, physiological brain aging is characterized by a loss of synaptic contacts and neuronal cell death that provokes age-dependent declines of sensory processing and motor control (Rossini et al., 2007; Boisgontier et al., 2012; Xifra-Porxas et al., 2019). Thus, it may be possible that age-related decrease in tactile sensibility and the ability to process sensory feedback (Cole et al., 1999) lead to declines in grip force control and increased brain activity within the motor-cortical network regardless of the task complexity. Following the compensation theory of aging (Reuter-Lorenz and Park, 2010), over-recruitment of cortical resources with aging appears to reflect a greater computational effort and oxygenation owing to compensatory patterns such as reorganization and redistribution of functional networks to counteract age-related structural and neurochemical changes in motor control systems (Bennett and Castiello, 1994; Mattay et al., 2002; Ward, 2006; Noble et al., 2011; Larivière et al., 2019). Nevertheless, the results of deteriorations in grip force control and higher hemodynamic brain activity in older subjects cannot only be associated with the grip force tasks. As we did not include an additional control task such as a pinch force task or similar, increased brain activity in healthy aging during movements may be a general result that might also occur in other motor tasks and not only during the FTT. Furthermore, we identified decreases in beta power during both FTT relative to the resting period, known as MRBD, which showed the typical attenuation of beta activity during voluntary movements. After completion, beta power is pronounced, which has been interpreted as increased inhibition to maintain the status quo of the motor system (Pfurtscheller et al., 2006; Koelewijn et al., 2008; Engel and Fries, 2010). However, previous studies showed that older adults exhibited significantly higher beta oscillations at rest as well as greater MRBD during dynamic muscle contractions than did younger controls (Xifra-Porxas et al., 2019). However, our results did not show any significant differences in baseline beta power between the two groups. Additionally, older adults exhibited significantly greater MRBD in SMC bilaterally during the easy static FTT rather than during the complex variable FTT.

Second, all participants showed significantly higher inaccuracies in grip force control during the complex FTT than during the easy FTT. While the performance of grip force control was affected by task complexity, the respective hemodynamic activity seems to be unaffected. This is in line with the findings of previous studies, which showed that hemodynamic responses for all motor-cortical regions and two different grip force execution tasks (20% and 40% of the maximum grip strength) were similar (Wriessnegger et al., 2017). In contrast, significant differences in hemodynamic activity owing to task complexity were detected during finger tapping (Holper et al., 2009) and hand squeezing tasks in young healthy participants (Cramer et al., 2002). Thus, task-specific factors other than task complexity seem to affect hemodynamic responses. Furthermore, we identified that complexity-related deteriorations in grip force control were correlated with increased left PMC beta activity in older adults, which differ significantly (*p* = 0.03) from the negative correlation of the younger adults. Therefore, the processing of visuomotor transformation and the integration of sensory feedback that underly precise grip force regulation (Hermsdörfer et al., 2003; Prodoehl et al., 2009) may be different between younger and older adults. In previous studies, older adults showed significantly higher absolute beta activity during movements than did younger adults (Heinrichs-Graham and Wilson, 2016) that correlated with the degree of motor deficits during a visually guided grip task (Rossiter et al., 2014a). Due to the close relationship of beta activity and motor-cortical GABAergic inhibition (Rossiter et al., 2014b; Xifra-Porxas et al., 2019), it is suggested that age-related increases in beta activity may lead to a reduced ability to control the inhibition within the motor control network during movements. In the complex FTT, where the grip force must be regulated and adapted continuously, high cognitive effort in motor planning is required. Thus, older adults might show increased use of the PMC/SMA (Leff et al., 2011) which represents a key structure in the motor-cortical network driving manual movements by promoting and suppressing brain activity (Grefkes et al., 2008a; Bönstrup et al., 2016).

In addition to the age-related and task-complexity effects on grip force control and brain activity, correlations of oxygenated hemoglobin and oscillatory beta activity were found. During the easy FTT, increases in oscillatory beta activity were related to increases in oxygenated hemoglobin. However, in a previous study, increases in hemodynamic activity were accompanied by decreases of alpha and beta activity during a motor task (Lachert et al., 2017). Also, increases of alpha power following 10 and 20 Hz tACS were accompanied by decreases in oxygenated hemoglobin (Berger et al., 2018). Although it is well known that increases in oxygenated hemoglobin (Strangman et al., 2002) and suppressions in beta activity (Rossiter et al., 2014b; Xifra-Porxas et al., 2019) represent the neural correlated underlying movements, the relationship between the two parameters and how they change depending on motor tasks and complexities is not yet fully understood. Thus, numerous questions regarding the correlation of electrical signals and vascular changes (e.g., co-localization/time lag of the correlated changes, consistency of changes in connectivity) remain.

In summary, the older adults showed significant deterioration in grip force control and greater hemodynamic activity during both the FTTs than did the young adults, suggesting a greater computational effort and oxygen supply during the motor tasks (Ward, 2006) which represent a general age-related compensatory mechanism of changes in the sensorimotor network (Noble et al., 2011; Larivière et al., 2019). In contrast, increases in left PMC beta activity that are related to deteriorations in grip force regulation in older adults are task-complexity-dependent, thus representing an increased cognitive effort during motor planning or a compensatory mechanism of deficits in processing the sensory feedback within the sensorimotor network. Nevertheless, the age-related changes in oscillatory and hemodynamic activity as well as their correlation do not show clear results that allow an unequivocal conclusion. Understanding the relationship between motor control and brain activity as well as between motor recovery and its underlying neuroplasticity, remain major challenges in neurorehabilitation (Bönstrup et al., 2018). Thus, uncovering the link between age- and disease-related impairments of motor control and changes in brain activity to identify objective biomarkers for neuroplastic changes and motor recovery is a future goal in neurorehabilitation (Bönstrup et al., 2018; Coscia et al., 2019). Based on this, individualized training protocols and interventions with new technologies such as BMI and NIBS could be developed, which enable sustainable rehabilitation and optimization of the outcome and effectiveness of motor recovery (Semprini et al., 2018; Steinberg et al., 2019; Berger et al., 2019a,b).

### Limitations

Although our findings regarding age- and task complexity-related changes in precise grip force regulation and brain activity were interesting, three limitations must be discussed and considered while designing future studies. First, the small number of subjects (*n* = 32) limits a clear and general conclusion. Referring to other neurophysiological studies (Noble et al., 2011; Xifra-Porxas et al., 2019), we focused on the methodological challenge of a combined EEG-fNIRS measurement during a motor task. In future studies, however, a higher sample size should be considered. Second, all participants were required to match the same target force in the visually guided FTT. Although our two groups do not differ in terms of MVC, previous studies have shown that grip strength declines with increasing age (Zammit et al., 2019) and after neurological diseases (Hermsdörfer et al., 2003). Thus, individual target forces depending on their own individual MVC can be considered in future FTT tasks to ensure that an optimal balance of support and challenge for each patient is achieved. However, different percentual forces ranging from 5 to 60% of the MVC were used in previous studies (Voelcker-Rehage and Alberts, 2005; Wriessnegger et al., 2017). Third, we had to focus on specific motor-cortical ROI because of the limited number of fNIRS channels and limited spatial resolution. Based on the knowledge that skilled grip force control depends on the intact communication of the entire sensorimotor network (Hermsdörfer et al., 2003) and because previous studies investigated motor-cortical areas using fNIRS during handgrip tasks (Wriessnegger et al., 2017), premotor and sensorimotor areas were chosen for investigation. Nevertheless, daily activities requiring visuomotor and sensorimotor integration (Vingerhoets, 2014) are based on a large neural network in which further cortical and subcortical areas are also involved (Vaillancourt et al., 2007; Wasson et al., 2010). Also, further analyses such as the coherence analysis between ROI to evaluate network communication and other recordings such as EMG on the performing arm to control muscle activity and to more precisely describe possible causes for age-related changes in grip force control would have added valuable further insights.

### Conclusions

In this study, we found evidence for age-related changes in grip force control and hemodynamic activity as well as correlations between grip force control owing to task complexity and beta activity that were different in the left PMC between younger and older adults. Assessing both motor precision and brain activity is essential for understanding how motor control, oscillatory, and hemodynamic activity within the motor-cortical network are related. This knowledge could contribute to the development of new therapeutic approaches to individualize and sustainably improve the treatment of motor impairments.

## Data Availability Statement

The raw data supporting the conclusions of this article will be made available by the authors, without undue reservation.

## Ethics Statement

The studies involving human participants were reviewed and approved by Local ethics committee of the Johannes Gutenberg-Universität Mainz. The participants provided their written informed consent to participate in this study.

## Author Contributions

AB: conception and execution of the research project, data acquisition, statistical analysis, interpretation, and manuscript writing. AB acts as the corresponding author. FS, FT, and MD: conception and execution of the research project and review and critique of the manuscript. All authors have read and approved the final version of the manuscript.

## Conflict of Interest

The authors declare that the research was conducted in the absence of any commercial or financial relationships that could be construed as a potential conflict of interest.

## References

[B1] Agbangla N. F. Audiffren M. Albinet C. T. (2017). Use of near-infrared spectroscopy in the investigation of brain activation during cognitive aging: a systematic review of an emerging area of research. Ageing Res. Rev. 38, 52–66. 10.1016/j.arr.2017.07.003 28755870

[B2] Bönstrup M. Schulz R. Feldheim J. Hummel F. C. Gerloff C. (2016). Dynamic causal modelling of EEG and fMRI to characterize network architectures in a simple motor task. NeuroImage 124, 498–508. 10.1016/j.neuroimage.2015.08.052 26334836

[B3] Bönstrup M. Schulz R. Schön G. Cheng B. Feldheim J. Thomalla G.. (2018). Parietofrontal network upregulation after motor stroke. NeuroImage Clin. 18, 720–729. 10.1016/j.nicl.2018.03.006 29876261 PMC5987870

[B4] Bennett K. M. Castiello U. (1994). Reach to grasp: changes with age. J. Gerontol. 49, P1–P7. 10.1093/geronj/49.1.p1 8282979

[B5] Berger A. Horst F. Müller S. Steinberg F. Doppelmayr M. (2019a). Current state and future prospects of EEG and fNIRS in robot-assisted gait rehabilitation: a brief review. Front. Hum. Neurosci. 13:172. 10.3389/fnhum.2019.00172 31231200 PMC6561323

[B6] Berger A. Horst F. Steinberg F. Thomas F. Müller-Eising C. Schöllhorn W. I.. (2019b). Increased gait variability during robot-assisted walking is accompanied by increased sensorimotor brain activity in healthy people. J. Neuroeng. Rehabil. 16:161. 10.1186/s12984-019-0636-3 31882008 PMC6935063

[B7] Berger A. Pixa N. H. Steinberg F. Doppelmayr M. (2018). Brain oscillatory and hemodynamic activity in a bimanual coordination task following transcranial alternating current stimulation (tACS): a combined EEG-fNIRS study. Front. Behav. Neurosci. 12:67. 10.3389/fnbeh.2018.00067 29720935 PMC5915568

[B8] Bock O. Steinberg F. (2012). Age-related deficits of manual grasping in a laboratory versus in an everyday-like setting. Ageing Res. 3:e7 10.4081/ar.2012.e7

[B9] Boisgontier M. P. Olivier I. Chenu O. Nougier V. (2012). Presbypropria: the effects of physiological ageing on proprioceptive control. Age 34, 1179–1194. 10.1007/s11357-011-9300-y 21850402 PMC3448996

[B10] Cohen J. (1988). Statistical Power Analysis for the Behavioral Sciences. 2nd Edn. Hillsdale, NJ: L. Erlbaum Associates.

[B11] Cole K. J. (1991). Grasp force control in older adults. J. Mot. Behav. 23, 251–258. 10.1080/00222895.1991.9942036 14766507

[B12] Cole K. J. Rotella D. L. Harper J. G. (1999). Mechanisms for age-related changes of fingertip forces during precision gripping and lifting in adults. J. Neurosci. 19, 3238–3247. 10.1523/JNEUROSCI.19-08-03238.1999 10191336 PMC6782297

[B13] Cope M. Delpy D. T. Reynolds E. O. Wray S. Wyatt J. van der Zee P. (1988). Methods of quantitating cerebral near infrared spectroscopy data. Adv. Exp. Med. Biol. 222, 183–189. 10.1007/978-1-4615-9510-6_21 3129910

[B14] Coscia M. Wessel M. J. Chaudary U. Millán J. D. R. Micera S. Guggisberg A.. (2019). Neurotechnology-aided interventions for upper limb motor rehabilitation in severe chronic stroke. Brain 142, 2182–2197. 10.1093/brain/awz181 31257411 PMC6658861

[B15] Cramer S. C. Weisskoff R. M. Schaechter J. D. Nelles G. Foley M. Finklestein S. P.. (2002). Motor cortex activation is related to force of squeezing. Hum. Brain Mapp. 16, 197–205. 10.1002/hbm.10040 12112762 PMC6871791

[B16] Dai T. H. Liu J. Z. Sahgal V. Brown R. W. Yue G. H. (2001). Relationship between muscle output and functional MRI-measured brain activation. Exp. Brain Res. 140, 290–300. 10.1007/s002210100815 11681304

[B17] Davis N. J. Tomlinson S. P. Morgan H. M. (2012). The role of β-frequency neural oscillations in motor control. J. Neurosci. 32, 403–404. 10.1523/JNEUROSCI.5106-11.2012 22238075 PMC6621073

[B18] Diermayr G. McIsaac T. L. Gordon A. M. (2011). Finger force coordination underlying object manipulation in the elderly—a mini-review. Gerontology 57, 217–227. 10.1159/000295921 20224251

[B19] Ehrsson H. H. Fagergren A. Jonsson T. Westling G. Johansson R. S. Forssberg H. (2000). Cortical activity in precision- versus power-grip tasks: an fMRI study. J. Neurophysiol. 83, 528–536. 10.1152/jn.2000.83.1.528 10634893

[B30] Eid M. Gollwitzer M. Schmitt M. (2015). Statistik und Forschungsmethoden: Mit Online-Materialien 5. korrigierte auflage. Weinheim: Beltz.

[B20] Engel A. K. Fries P. (2010). Beta-band oscillations—signalling the status quo? Curr. Opin. Neurobiol. 20, 156–165. 10.1016/j.conb.2010.02.015 20359884

[B21] Flanagan J. R. Wing A. M. (1995). The stability of precision grip forces during cyclic arm movements with a hand-held load. Exp. Brain Res. 105, 455–464. 10.1007/BF00233045 7498399

[B22] Gölz C. Voelcker-Rehage C. Mora K. Reuter E.-M. Godde B. Dellnitz M.. (2018). Improved neural control of movements manifests in expertise-related differences in force output and brain network dynamics. Front. Physiol. 9:1540. 10.3389/fphys.2018.01540 30519188 PMC6258820

[B23] Gassert R. Dietz V. (2018). Rehabilitation robots for the treatment of sensorimotor deficits: a neurophysiological perspective. J. Neuroeng. Rehabil. 15:46. 10.1186/s12984-018-0383-x 29866106 PMC5987585

[B24] Gratton G. Coles M. G. H. Donchin E. (1983). A new method for off-line removal of ocular artifact. Electroencephalogr. Clin. Neurophysiol. 55, 468–484. 10.1016/0013-4694(83)90135-9 6187540

[B25] Grefkes C. Eickhoff S. B. Nowak D. A. Dafotakis M. Fink G. R. (2008a). Dynamic intra- and interhemispheric interactions during unilateral and bilateral hand movements assessed with fMRI and DCM. NeuroImage 41, 1382–1394. 10.1016/j.neuroimage.2008.03.048 18486490

[B26] Grefkes C. Nowak D. A. Eickhoff S. B. Dafotakis M. Küst J. Karbe H.. (2008b). Cortical connectivity after subcortical stroke assessed with functional magnetic resonance imaging. Ann. Neurol. 63, 236–246. 10.1002/ana.21228 17896791

[B27] Haggard P. Wing A. (1995). Coordinated responses following mechanical perturbation of the arm during prehension. Exp. Brain Res. 102, 483–494. 10.1007/BF00230652 7737394

[B28] Hamacher D. Herold F. Wiegel P. Hamacher D. Schega L. (2015). Brain activity during walking: a systematic review. Neurosci. Biobehav. Rev. 57, 310–327. 10.1016/j.neubiorev.2015.08.002 26306029

[B29] Heinrichs-Graham E. Wilson T. W. (2016). Is an absolute level of cortical beta suppression required for proper movement? Magnetoencephalographic evidence from healthy aging. NeuroImage 134, 514–521. 10.1016/j.neuroimage.2016.04.032 27090351 PMC4912897

[B31] Hermsdörfer J. Hagl E. Nowak D. A. Marquardt C. (2003). Grip force control during object manipulation in cerebral stroke. Clin. Neurophysiol. 114, 915–929. 10.1016/s1388-2457(03)00042-7 12738439

[B32] Hermsdörfer J. Marquardt C. Philipp J. Zierdt A. Nowak D. Glasauer S.. (2000). Moving weightless objects. Grip force control during microgravity. Exp. Brain Res. 132, 52–64. 10.1007/s002219900325 10836635

[B33] Herold F. Wiegel P. Scholkmann F. Thiers A. Hamacher D. Schega L. (2017). Functional near-infrared spectroscopy in movement science: a systematic review on cortical activity in postural and walking tasks. Neurophotonics 4:041403. 10.1117/1.NPh.4.4.041403 28924563 PMC5538329

[B34] Holper L. Biallas M. Wolf M. (2009). Task complexity relates to activation of cortical motor areas during uni- and bimanual performance: a functional NIRS study. NeuroImage 46, 1105–1113. 10.1016/j.neuroimage.2009.03.027 19306929

[B35] Hutchinson S. Kobayashi M. Horkan C. M. Pascual-Leone A. Alexander M. P. Schlaug G. (2002). Age-related differences in movement representation. NeuroImage 17, 1720–1728. 10.1006/nimg.2002.1309 12498746

[B36] Ishii R. Canuet L. Aoki Y. Hata M. Iwase M. Ikeda S.. (2017). Healthy and pathological brain aging: from the perspective of oscillations, functional connectivity and signal complexity. Neuropsychobiology 75, 151–161. 10.1159/000486870 29466802

[B37] Jurcak V. Tsuzuki D. Dan I. (2007). 10/20, 10/10 and 10/5 systems revisited: their validity as relative head-surface-based positioning systems. NeuroImage 34, 1600–1611. 10.1016/j.neuroimage.2006.09.024 17207640

[B38] Kim J.-H. Lee Y.-S. Lee J.-J. Song H.-J. Yoo D.-S. Lee H. J.. (2010). Functional magnetic resonance imaging reveals age-related alterations to motor networks in weighted elbow flexion-extension movement. Neurol. Res. 32, 995–1001. 10.1179/016164110X12670144737693 20433774

[B39] Kiyama S. Kunimi M. Iidaka T. Nakai T. (2014). Distant functional connectivity for bimanual finger coordination declines with aging: an fMRI and SEM exploration. Front. Hum. Neurosci. 8:251. 10.3389/fnhum.2014.00251 24795606 PMC4007017

[B40] Koelewijn T. van Schie H. T. Bekkering H. Oostenveld R. Jensen O. (2008). Motor-cortical beta oscillations are modulated by correctness of observed action. NeuroImage 40, 767–775. 10.1016/j.neuroimage.2007.12.018 18234516

[B41] Koenraadt K. L. M. Roelofsen E. G. J. Duysens J. Keijsers N. L. W. (2014). Cortical control of normal gait and precision stepping: an fNIRS study. NeuroImage 85, 415–422. 10.1016/j.neuroimage.2013.04.070 23631980

[B42] Lachert P. Janusek D. Pulawski P. Liebert A. Milej D. Blinowska K. J. (2017). Coupling of oxy- and deoxyhemoglobin concentrations with EEG rhythms during motor task. Sci. Rep. 7:15414. 10.1038/s41598-017-15770-2 29133861 PMC5684354

[B43] Larivière S. Xifra-Porxas A. Kassinopoulos M. Niso G. Baillet S. Mitsis G. D.. (2019). Functional and effective reorganization of the aging brain during unimanual and bimanual hand movements. Hum. Brain Mapp. 40, 3027–3040. 10.1002/hbm.24578 30866155 PMC6548623

[B44] Leff D. R. Orihuela-Espina F. Elwell C. E. Athanasiou T. Delpy D. T. Darzi A. W.. (2011). Assessment of the cerebral cortex during motor task behaviours in adults: a systematic review of functional near infrared spectroscopy (fNIRS) studies. NeuroImage 54, 2922–2936. 10.1016/j.neuroimage.2010.10.058 21029781

[B45] Makeig S. Gramann K. Jung T.-P. Sejnowski T. J. Poizner H. (2009). Linking brain, mind and behavior. Int. J. Psychophysiol. 73, 95–100. 10.1016/j.ijpsycho.2008.11.008 19414039 PMC2796545

[B46] Massy-Westropp N. M. Gill T. K. Taylor A. W. Bohannon R. W. Hill C. L. (2011). Hand grip strength: age and gender stratified normative data in a population-based study. BMC Res. Notes 4:127. 10.1186/1756-0500-4-127 21492469 PMC3101655

[B47] Mattay V. S. Fera F. Tessitore A. Hariri A. R. Das S. Callicott J. H.. (2002). Neurophysiological correlates of age-related changes in human motor function. Neurology 58, 630–635. 10.1212/wnl.58.4.630 11865144

[B48] Minati L. Grisoli M. Bruzzone M. G. (2007). MR spectroscopy, functional MRI and diffusion-tensor imaging in the aging brain: a conceptual review. J. Geriatr. Psychiatry Neurol. 20, 3–21. 10.1177/0891988706297089 17341766

[B49] Naccarato M. Calautti C. Jones P. S. Day D. J. Carpenter T. A. Baron J.-C. (2006). Does healthy aging affect the hemispheric activation balance during paced index-to-thumb opposition task? an fMRI study. NeuroImage 32, 1250–1256. 10.1016/j.neuroimage.2006.05.003 16806984

[B50] Noble J. W. Eng J. J. Kokotilo K. J. Boyd L. A. (2011). Aging effects on the control of grip force magnitude: an fMRI study. Exp. Gerontol. 46, 453–461. 10.1016/j.exger.2011.01.004 21296649 PMC3096652

[B51] Nowak D. A. Hermsdörfer J. Philipp J. Marquardt C. Glasauer S. Mai N. (2001). Effects of changing gravity on anticipatory grip force control during point-to-point movements of a hand-held object. Motor Control 5, 231–253. 10.1123/mcj.5.3.231 11438763

[B52] Obrig H. Villringer A. (2003). Beyond the visible—imaging the human brain with light. J. Cereb. Blood Flow Metab. 23, 1–18. 10.1097/01.WCB.0000043472.45775.29 12500086

[B300] Oldfield R. C. (1971). The assessment and analysis of handedness: the Edinburgh inventory. Neuropsychologia 9, 97–113. 10.1016/0028-3932(71)90067-4 5146491

[B53] Parry R. Macias Soria S. Pradat-Diehl P. Marchand-Pauvert V. Jarrassé N. Roby-Brami A. (2019). Effects of hand configuration on the grasping, holding and placement of an instrumented object in patients with hemiparesis. Front. Neurol. 10:240. 10.3389/fneur.2019.00240 30941091 PMC6433942

[B54] Pfurtscheller G. Leeb R. Keinrath C. Friedman D. Neuper C. Guger C.. (2006). Walking from thought. Brain Res. 1071, 145–152. 10.1016/j.brainres.2005.11.083 16405926

[B55] Pollok B. Boysen A.-C. Krause V. (2015). The effect of transcranial alternating current stimulation (tACS) at alpha and beta frequency on motor learning. Behav. Brain Res. 293, 234–240. 10.1016/j.bbr.2015.07.049 26225845

[B56] Prodoehl J. Corcos D. M. Vaillancourt D. E. (2009). Basal ganglia mechanisms underlying precision grip force control. Neurosci. Biobehav. Rev. 33, 900–908. 10.1016/j.neubiorev.2009.03.004 19428499 PMC2684813

[B57] Rand M. K. Shimansky Y. Stelmach G. E. Bloedel J. R. (2004). Adaptation of reach-to-grasp movement in response to force perturbations. Exp. Brain Res. 154, 50–65. 10.1007/s00221-003-1637-8 14530893

[B58] Reuter-Lorenz P. A. Park D. C. (2010). Human neuroscience and the aging mind: a new look at old problems. J. Gerontol. B Psychol. Sci. Soc. Sci. 65, 405–415. 10.1093/geronb/gbq035 20478901 PMC2883872

[B59] Rossini P. M. Rossi S. Babiloni C. Polich J. (2007). Clinical neurophysiology of aging brain: from normal aging to neurodegeneration. Prog. Neurobiol. 83, 375–400. 10.1016/j.pneurobio.2007.07.010 17870229

[B60] Rossiter H. E. Boudrias M.-H. Ward N. S. (2014a). Do movement-related beta oscillations change after stroke? J. Neurophysiol. 112, 2053–2058. 10.1152/jn.00345.2014 25080568 PMC4274928

[B61] Rossiter H. E. Davis E. M. Clark E. V. Boudrias M.-H. Ward N. S. (2014b). Beta oscillations reflect changes in motor cortex inhibition in healthy ageing. NeuroImage 91, 360–365. 10.1016/j.neuroimage.2014.01.012 24440529 PMC3988925

[B62] Sassaroli A. Fantini S. (2004). Comment on the modified Beer-Lambert law for scattering media. Phys. Med. Biol. 49, N255–N257. 10.1088/0031-9155/49/14/n07 15357206

[B63] Schnitzler A. Gross J. (2005). Normal and pathological oscillatory communication in the brain. Nat. Rev. Neurosci. 6, 285–296. 10.1038/nrn1650 15803160

[B64] Semprini M. Laffranchi M. Sanguineti V. Avanzino L. De Icco R. De Michieli L.. (2018). Technological approaches for neurorehabilitation: from robotic devices to brain stimulation and beyond. Front. Neurol. 9:212. 10.3389/fneur.2018.00212 29686644 PMC5900382

[B65] Steinberg F. Pixa N. H. Fregni F. (2019). A review of acute aerobic exercise and transcranial direct current stimulation effects on cognitive functions and their potential synergies. Front. Hum. Neurosci. 12:534. 10.3389/fnhum.2018.00534 30687048 PMC6336823

[B66] Strangman G. Culver J. P. Thompson J. H. Boas D. A. (2002). A quantitative comparison of simultaneous BOLD fMRI and NIRS recordings during functional brain activation. NeuroImage 17, 719–731. 10.1006/nimg.2002.1227 12377147

[B67] Suzuki M. Miyai I. Ono T. Oda I. Konishi I. Kochiyama T.. (2004). Prefrontal and premotor cortices are involved in adapting walking and running speed on the treadmill: an optical imaging study. NeuroImage 23, 1020–1026. 10.1016/j.neuroimage.2004.07.002 15528102

[B68] Taniguchi M. Kato A. Fujita N. Hirata M. Tanaka H. Kihara T.. (2000). Movement-related desynchronization of the cerebral cortex studied with spatially filtered magnetoencephalography. NeuroImage 12, 298–306. 10.1006/nimg.2000.0611 10944412

[B69] Teo W.-P. Muthalib M. Yamin S. Hendy A. M. Bramstedt K. Kotsopoulos E.. (2016). Does a combination of virtual reality, neuromodulation and neuroimaging provide a comprehensive platform for neurorehabilitation?—A narrative review of the literature. Front. Hum. Neurosci. 10:284. 10.3389/fnhum.2016.00284 27445739 PMC4919322

[B70] Vaillancourt D. E. Yu H. Mayka M. A. Corcos D. M. (2007). Role of the basal ganglia and frontal cortex in selecting and producing internally guided force pulses. NeuroImage 36, 793–803. 10.1016/j.neuroimage.2007.03.002 17451971 PMC1950146

[B71] Vingerhoets G. (2014). Contribution of the posterior parietal cortex in reaching, grasping and using objects and tools. Front. Psychol. 5:151. 10.3389/fpsyg.2014.00151 24634664 PMC3942635

[B72] Vitorio R. Stuart S. Rochester L. Alcock L. Pantall A. (2017). fNIRS response during walking—artefact or cortical activity? A systematic review. Neurosci. Biobehav. Rev. 83, 160–172. 10.1016/j.neubiorev.2017.10.002 29017917

[B73] Voelcker-Rehage C. Alberts J. L. (2005). Age-related changes in grasping force modulation. Exp. Brain Res. 166, 61–70. 10.1007/s00221-005-2342-6 16096780

[B74] Wach C. Krause V. Moliadze V. Paulus W. Schnitzler A. Pollok B. (2013). The effect of 10 Hz transcranial alternating current stimulation (tACS) on corticomuscular coherence. Front. Hum. Neurosci. 7:511. 10.3389/fnhum.2013.00511 24009573 PMC3756226

[B75] Ward N. S. (2006). Compensatory mechanisms in the aging motor system. Ageing Res. Rev. 5, 239–254. 10.1016/j.arr.2006.04.003 16905372

[B76] Wasson P. Prodoehl J. Coombes S. A. Corcos D. M. Vaillancourt D. E. (2010). Predicting grip force amplitude involves circuits in the anterior basal ganglia. NeuroImage 49, 3230–3238. 10.1016/j.neuroimage.2009.11.047 19944767 PMC2818558

[B77] Wriessnegger S. C. Kirchmeyr D. Bauernfeind G. Müller-Putz G. R. (2017). Force related hemodynamic responses during execution and imagery of a hand grip task: a functional near infrared spectroscopy study. Brain Cogn. 117, 108–116. 10.1016/j.bandc.2017.06.010 28673464

[B78] Xifra-Porxas A. Niso G. Larivière S. Kassinopoulos M. Baillet S. Mitsis G. D.. (2019). Older adults exhibit a more pronounced modulation of beta oscillations when performing sustained and dynamic handgrips. NeuroImage 201:116037. 10.1016/j.neuroimage.2019.116037 31330245 PMC6765431

[B79] Xu Y. Graber H. L. Barbour R. L. (2014). “nirsLAB: a computing environment for fNIRS neuroimaging data analysis,” in Proceedings Biomedical Optics 2014 (Miami, FL: Optical Society of America), Paper BM3A.1.

[B80] Zaepffel M. Trachel R. Kilavik B. E. Brochier T. (2013). Modulations of EEG beta power during planning and execution of grasping movements. PLoS One 8:e60060. 10.1371/journal.pone.0060060 23555884 PMC3605373

[B81] Zammit A. R. Robitaille A. Piccinin A. M. Muniz-Terrera G. Hofer S. M. (2019). Associations between aging-related changes in grip strength and cognitive function in older adults: a systematic review. J. Gerontol. A Biol. Sci. Med. Sci. 74, 519–527. 10.1093/gerona/gly046 29528368 PMC6417444

